# Does Tourniquet Use Impact Early Patient Outcomes in Total Knee Arthroplasty?

**DOI:** 10.7759/cureus.73379

**Published:** 2024-11-10

**Authors:** Mohamed Elbeshbeshy, Muhammad Saad Azhar, Muhammad Luqman, Ahmad Sabahuddin, Oladimeji Bashir, Ahmed Y Saber, James Parker, Osman Riaz, Timothy G McWilliams, Adeel Aqil

**Affiliations:** 1 Trauma and Orthopaedics, Calderdale and Huddersfield NHS Foundation Trust, Huddersfield, GBR

**Keywords:** blood loss, bone cement penetration, cement penetration, functional outcomes, pain, tka, total knee arthroplasty, tourniquet

## Abstract

Introduction

Tourniquets are commonly used when performing total knee arthroplasty (TKA) to reduce intra-operative blood loss, improve surgical field visibility, and potentially improve cement penetration during prosthesis implantation. However, they may be associated with increased thigh pain, postoperative opiate use, and longer lengths of hospital stay.

Methods

Retrospectively collected data was obtained from our institution's electronic patient records and database. We compared data between those patients receiving a TKA with or without tourniquet use. Our primary outcome measure was cement penetration on immediate postoperative X-rays. Secondary outcome measures included the need for opiate analgesia, blood loss, need for transfusion, and length of hospital stay.

Results

There were 285 patients in this study, with 170 patients undergoing TKA with a tourniquet and 115 patients without a tourniquet. There was a significantly better median total cement penetration as measured on combined anteroposterior (AP) and lateral radiographs in the tourniquet group (39.14 vs 33.3mm, U=6991, z=-4.08, p<0.01). There was a statistically lower drop in hemoglobin levels when tourniquets were used (-13.4 (SD=8.6) vs -15.5 (SD=8.8), p=0.03). However, there were no cases in our series where patients required a transfusion. There was no significant difference in opiate analgesia requirements between tourniquet and non-tourniquet groups following surgery, 115 (68%) vs 69 (60%); X^2 ^(1,285)=1.75, p=0.19. The median length of stay following surgery was slightly longer in the tourniquet group (2.5 vs 2.8 days); however, the Mann-Whitney U test indicated that this difference was not significant (U=9076, z=-0.70, p=0.31).

Conclusion

The use of a tourniquet was associated with significantly improved bone cement penetration as measured on postoperative AP and lateral radiographs. However, the clinical relevance of this in terms of implant survival remains controversial. Tourniquet use was also significantly associated with lower blood loss, but this did not translate into a lower need for transfusions. The use of a tourniquet was not associated with increased analgesia requirements following surgery or significantly longer lengths of stay.

## Introduction

Total knee arthroplasty (TKA) is a cost-effective intervention in patients with knee osteoarthritis with high patient satisfaction and a significant improvement in quality of life [1]. With advancements in prosthesis design and improvements in patient-reported outcomes, over 100,000 TKAs are performed annually in the UK, with over one million performed globally [2].

The use of a tourniquet helps minimize blood loss, provide a clear operating field, and obtain dry bone surfaces for better cement interdigitation [3]. Some believe the latter is closely related to implant survival as it provides mechanical stability, better load distribution, and reduced micromotion. Theoretically, this should reduce the risk of aseptic loosening, which is the most common cause of TKA failure necessitating revision surgery [4,5].

The interference of blood between the bone cement interface may affect the strength of the fixation as it may not allow optimal cement interdigitation [6]. The effect of tourniquets on long-term implant survival is inconclusive, with small studies that publish short and medium-term follow-up data [7-9]. A more recent retrospective study reviewed data from the National Joint Registry from the year 2013 and concluded that at 13 years following surgery, all-cause revision between the two groups did not differ statistically [10]. However, this study had several confounding factors, missing data, and a small sample size of patients who had surgery without a tourniquet. Due to these reasons, tourniquets are routinely used in TKA, which was demonstrated by a British Association of Knee Surgeons (BASK) survey showing that 90% of surgeons utilize them routinely [11].

However, the use of tourniquets comes at a risk. Ahmed et al. performed a meta-analysis of the literature incorporating randomized control trials and concluded that tourniquets lead to a shorter surgical time, presumably due to a clear operating field, but carried risks of adverse events including reduced range of movement, deep vein thrombosis (DVT), pulmonary embolism (PE), infection and re-operation [12].

The benefits of tourniquet use can be balanced with potential complications by limiting their use to part of the procedure. Olivecrona et al. showed the average tourniquet time for TKA until wound closure was achieved was 80 minutes compared with 69 minutes if released after cementation alone. This would allow for better preparation for bone surfaces and cement interdigitation while reducing some of the risks shown in the literature. In their study, they showed complication rates were higher in the longer-use group [13].

The question of whether a tourniquet improves cement penetration and interdigitation remains controversial. Sun et al. showed from a meta-analysis that tourniquet use does increase bone cement penetration [14]. Conversely, Yao et al. argued from a similarly designed study that there is no increase in bone cement penetration with tourniquet use [15].

The use of tourniquets in TKA is evidently a topic of ongoing debate, given the opposing evidence and studies with a low volume number of included patients. The aim of our study was to contribute evidence from our local population by determining whether there is an improvement in bone cement penetration with the use of a tourniquet.

## Materials and methods

This retrospective study analyzed data obtained from our institution's electronic patient records (EPR) and database, focusing on 285 patients who underwent total knee arthroplasty (TKA). The objective was to compare outcomes between patients who had the procedure performed with the use of a tourniquet and those who did not. To ensure consistency and reduce variability, only patients who received a single brand of implant, specifically the P.F.C.™ Sigma® (DePuy Synthes, Raynham, US), and had their surgeries conducted using a standardized cementation technique by experienced hip and knee arthroplasty surgeons were included. The primary outcome measure was the extent of cement penetration observed on immediate postoperative X-rays. To correct the magnification error, we calculated the ratio of the actual tibial implant keel diameter to the diameter measured on the X-rays, thereby determining the magnification error, which was then used to adjust the measurements of cement penetration.

Cement penetration was measured in seven specific areas around the tibial keel on the anteroposterior (AP) radiograph of the knee and in three areas on the lateral radiograph of the knee, following the guidelines set by the Knee Society Roentgenographic Evaluation System (KSRES) [16]**.** These measurements were critical in determining the total cement penetration, which was calculated by summing the measurements from zones one to seven on AP view and zones one to three on lateral radiographs. Thus, the primary outcome measures included total AP cement penetration, total lateral cement penetration, and combined cement penetration from both AP and lateral radiographs.

**Figure 1:**
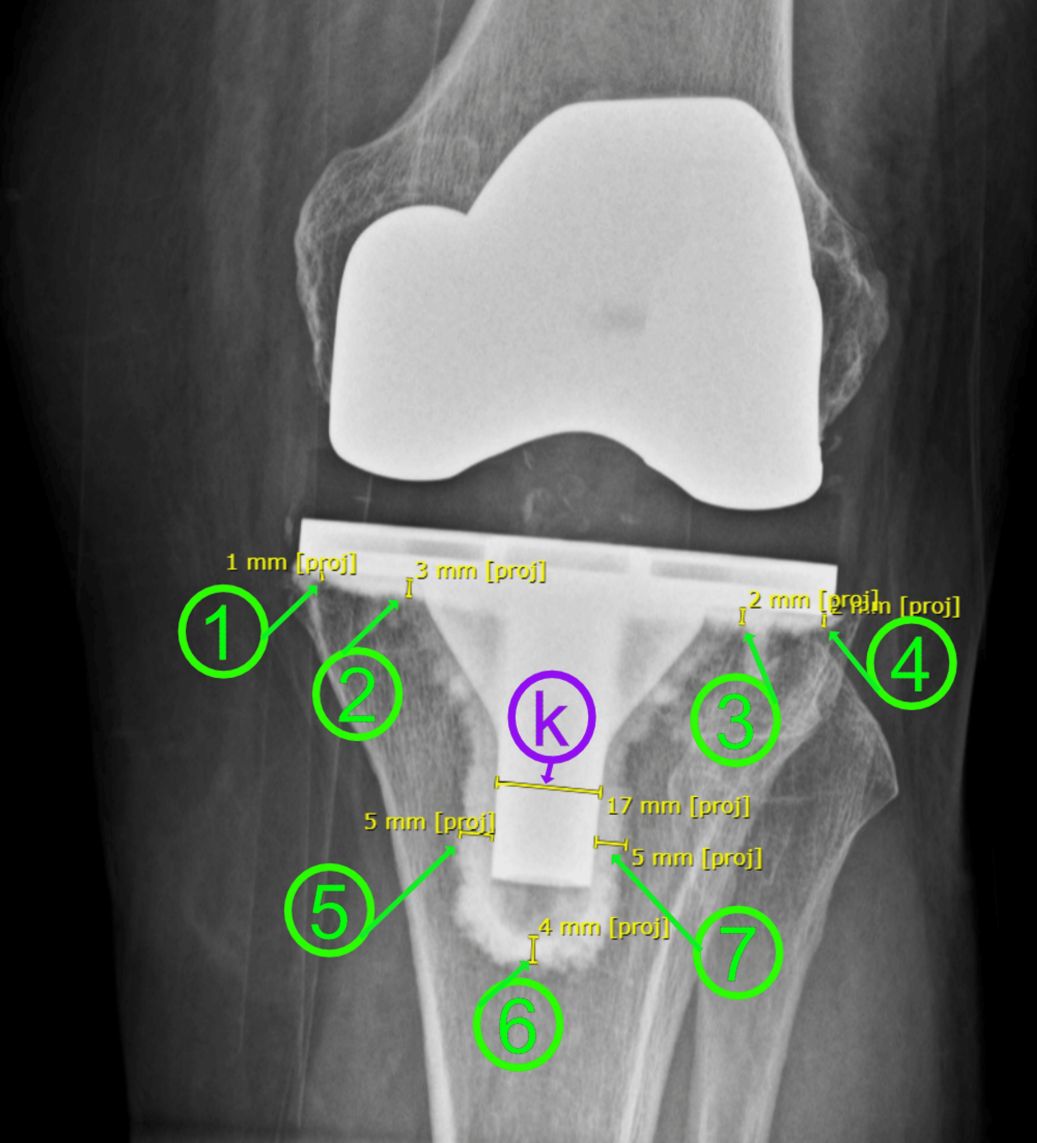
Measurement of cement penetrations on AP knee X-rays The green circles containing numbers 1-7 indicate the measured respective zones from zone 1 to zone 7. The purple circle containing the letter "k" indicates the keel diameter measurement. AP - anteroposterior Based on the Knee Society Roentgenographic Evaluation System [16].

**Figure 2:**
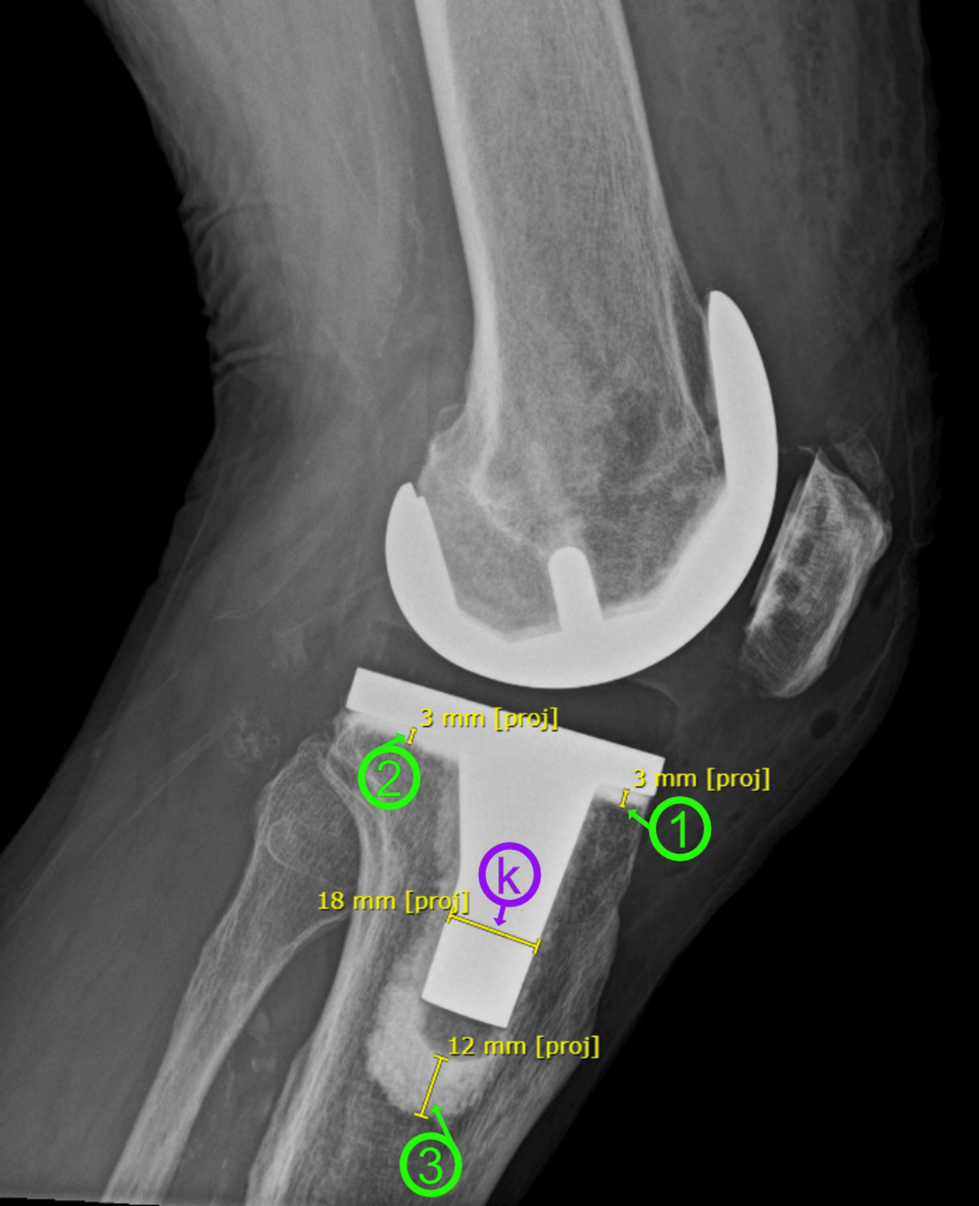
Measurement of cement penetrations on lateral knee X-rays The green circles containing numbers 1-3 indicate the measured respective zones from zone 1 to zone 3. The purple circle containing the letter "k" indicates the keel diameter measurement. Based on the Knee Society Roentgenographic Evaluation System [16].

Radiographic measurements were conducted immediately postoperatively. The actual tibial implant keel diameter served as a reference for calibrating the X-ray measurements. The magnification error was calculated by comparing the actual diameter with the measured diameter on the X-ray, and this error was corrected to ensure accurate measurement of cement penetration (Figures 1, 2). The cement penetration in the defined zones around the tibial keel on both AP and lateral radiographs was carefully measured, and the total cement penetration was computed by summing these values. Combined cement penetration was determined by adding the total measurements from both radiographs (Figures 1, 2).

In addition to the primary outcome measures, we examined several secondary outcome measures. These included the need for opiate analgesia postoperatively, the amount of blood loss during surgery, the requirement for blood transfusions postoperatively, and the length of hospital stay following the surgery. To identify potential risk factors contributing to variability in cement penetration, univariate analyses were performed, examining variables such as patient age and sex.

The statistical analysis began with the use of the Kolmogorov-Smirnov test to assess whether the continuous data approximated a normal distribution. Depending on the results of this test, appropriate statistical methods were employed for subsequent analyses. For parametric data, which were normally distributed, t-tests were used to compare means between groups. For nonparametric data, which were not normally distributed, the Mann-Whitney U test was employed for continuous variables, and the Chi-square test was used for categorical variables.

The study design ensured a rigorous approach to data collection and analysis. By standardizing the implant type and cementation technique and applying meticulous statistical analyses, we aimed to reduce potential biases and confounding factors. This methodological rigor was intended to ensure that the findings regarding the impact of tourniquet use on cement penetration and other clinical outcomes were robust and reliable.

The results of the analyses were interpreted in the context of existing literature and clinical practice. The findings on cement penetration were compared with known benchmarks and standards from previous studies. The impact of tourniquet use on secondary outcomes was evaluated in light of clinical guidelines and patient care protocols. The potential implications of the findings for surgical practice and patient outcomes were discussed, with recommendations for future research and practice improvements.

## Results

In this study involving 285 patients, 170 underwent TKA with a tourniquet, while 115 patients had the procedure performed without a tourniquet. Table 1 summarises the main outcome statistics.

**Table 1:** Descriptive and inferential statistics for age, cement penetrations, length of hospital stay, and hemoglobin change AP - anteroposterior

	Tourniquet (TQ)	N	Mean	Median	SD	IQR	Min	Max	Test	Statistics	p
Age (years)	TQ used	170	70.65	70	8.18	11	49	95	Mann-Whitney U	9297	0.48
	TQ not used	115	71.21	73	7.95	10	41	89			
Total AP cement penetration (mm)	TQ used	170	25.56	25.44	5.96	7.19	10.3	57.67	Mann-Whitney U	6493	<0.001
	TQ not used	115	22.36	21.63	8.04	8.71	5.41	55.8			
Total lateral cement penetration (mm)	TQ used	170	14.63	13.75	4.89	5.65	5.91	48.55	Mann-Whitney U	8074	0.013
	TQ not used	115	13.61	12.81	6.36	7.73	3.7	39.3			
Total AP and lateral cement penetration	TQ used	170	40.18	39.14	10.03	10.78	17.61	106.22	Mann-Whitney U	6991	<0.001
	TQ not used	115	35.97	33.34	13.88	15.1	11.88	95.1			
Length of stay (days)	TQ used	170	3.46	2.8	2.62	1.98	1	27.4	Mann-Whitney U	9076	0.31
	TQ not used	115	3.04	2.5	1.49	1.2	1.3	8.5			
Hemoglobin change (g/L)	TQ used	170	-13.37	-13	8.61	12	-36	9	Student t-test	2.024	0.03
	TQ not used	115	-15.5	-16	8.82	12	-45	6			

**Figure 3:**
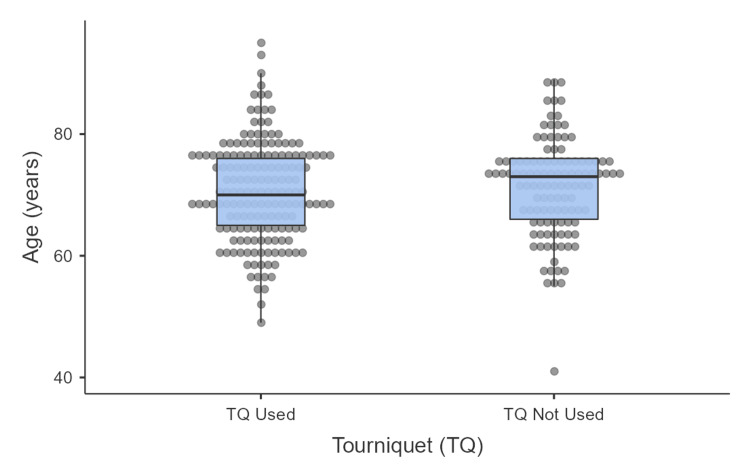
Age difference between the tourniquet and non-tourniquet groups

The median age of the entire cohort was 71 years, with a range extending from 41 to 95 years. A comparison of the median ages between the tourniquet group (70 years) and the non-tourniquet group (73 years) revealed no statistically significant difference, as indicated by a Mann-Whitney U test (U=9297.5, z=-0.70, p=0.48; Figure 3).

The tourniquet group experienced an average tourniquet pressure of 291 mmHg, with a range between 250 and 300 mmHg. The duration of surgery was similar between the two groups, with the tourniquet group having an average surgery time of 84 minutes compared to 83 minutes in the non-tourniquet group. This difference was not statistically significant (U=8745, z=-1.51, p=0.13).

Radiographic analysis of cement penetration, after correction for magnification errors, showed an average penetration of 24.3 mm (SD=7.04) on AP radiographs and 14.2 mm (SD=5.5) on lateral radiographs. The total average cement penetration, combining both AP and lateral measurements, was 38.4 mm (SD=11.90).

**Figure 4:**
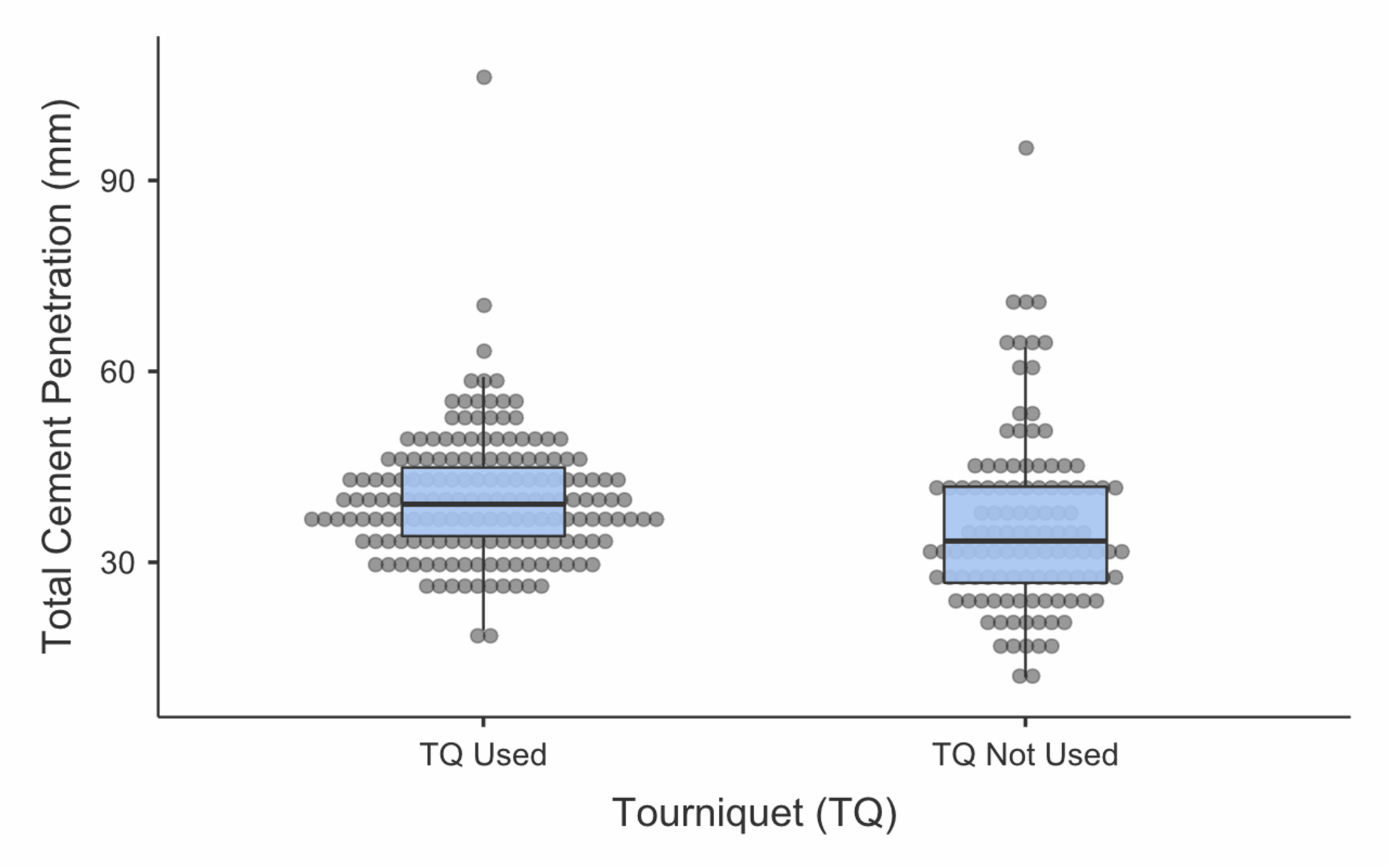
Difference in the total AP and lateral cement penetration between the tourniquet and non-tourniquet groups AP - anteroposterior

The tourniquet group demonstrated significantly better median total cement penetration on AP radiographs compared to the non-tourniquet group (25.44 mm vs 21.62 mm, U=6493, z=-4.81, p<0.01). Similarly, the tourniquet group also had superior median total cement penetration on lateral radiographs (13.75 mm vs 12.81 mm, U=8073, z=-2.49, p=0.01). When considering combined AP and lateral radiographs, the tourniquet group again showed significantly better median total cement penetration (39.14 mm vs 33.34 mm, U=6991, z=-4.08, p<0.001; Figure 4).

**Figure 5:**
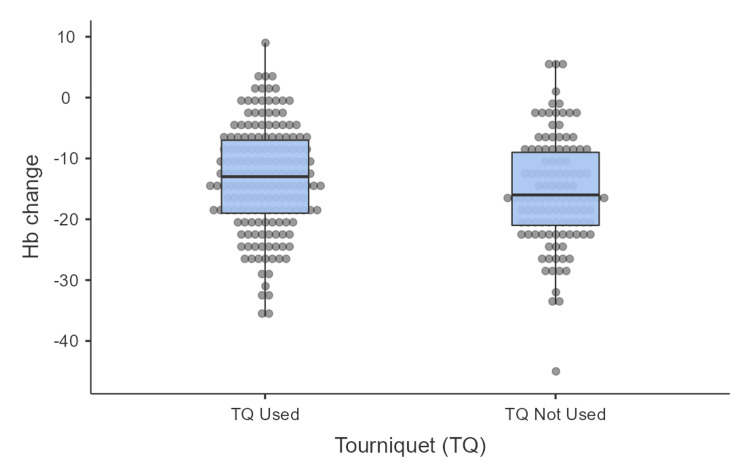
Difference in hemoglobin level change between the tourniquet and non-tourniquet groups

The average change in hemoglobin (HB) levels after surgery was 14.2 g/dL. There was a statistically lower drop in hemoglobin levels in the tourniquet group compared to the non-tourniquet group (-13.4 g/dL (SD=8.6) vs -15.5 g/dL (SD=8.8), p=0.03; Figure 5). Despite these differences, no patients required a blood transfusion in either group.

**Figure 6:**
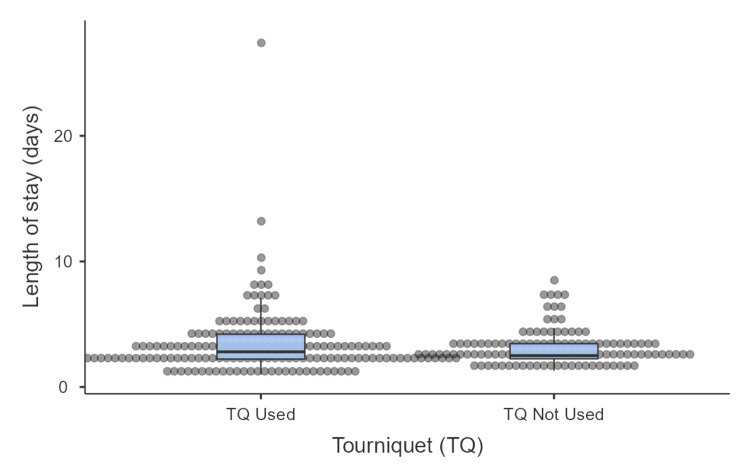
Difference in length of hospital stay between the tourniquet and non-tourniquet groups

Postoperative hospital stays, measured as the median length of stay, was 2.5 days across all patients, ranging from one to 27 days. The tourniquet group had a slightly longer median stay (2.5 days) compared to the non-tourniquet group (2.8 days). However, this difference was not statistically significant (U=9076, z=-0.70, p=0.31; Figure 6).

**Figure 7:**
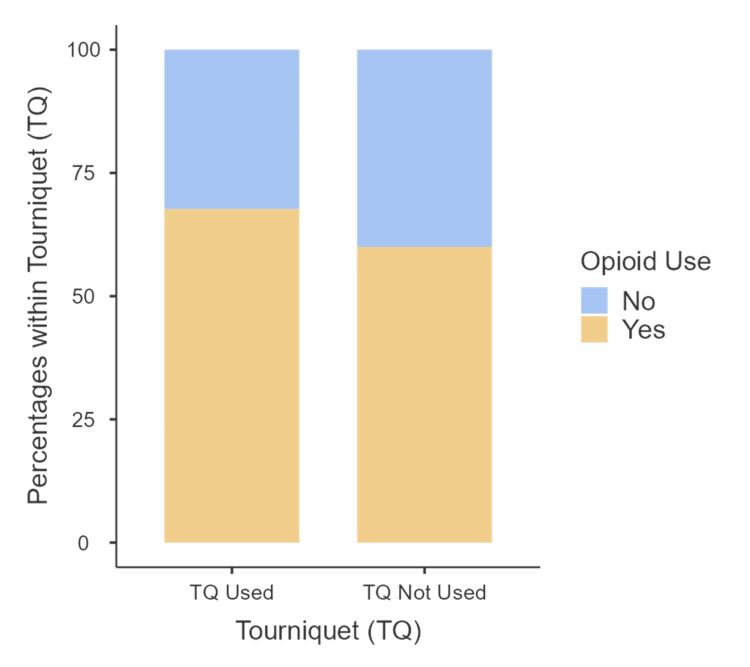
Percentage of opioid use within each of the tourniquet and non-tourniquet groups

Within the early postoperative period, 184 patients (65%) required opiate analgesia. There was no significant difference in the requirement for opiate analgesia post-surgery between the two groups, with 115 (68%) patients in the tourniquet group and 69 (60%) patients in the non-tourniquet group needing opiates (χ^2 ^(1,285)=1.75, p=0.19; Figure 7).

## Discussion

There has been a year-on-year increase in the number of TKAs performed in the UK based on NJR data, and the trend is likely to continue to rise [17]. This will inevitably lead to a higher number of revisions being required, mostly due to failure from aseptic loosening [18]. The cost of a revision TKA is generally significantly higher than that of a primary TKA due to the complexity of the procedure, the need for specialized implants, and increased hospital stay [19].

Aseptic loosening is the most common reason requiring revision, and it is therefore of vital importance to reduce this complication [4,5]. Central to addressing this issue is the achievement of effective bone cement penetration during surgery, which plays a potentially pivotal role in establishing a durable bone-cement interface critical for implant stability and longevity [18].

The importance of adequate cement penetration in reducing the risk of aseptic loosening has been underscored in the orthopedic literature. Bert et al. emphasized that optimal cementing techniques significantly influence the success and longevity of knee implants [20]. A deeper infiltration of cement into bone trabeculae enhances mechanical interlock, reduces micromotion, and improves load transfer, all crucial factors in preventing implant failure [4]. A recent retrospective study by Sasaki et al. emphasized the relationship between improved cement penetration and long-term survivability of the implant [21].

Better bone penetration and interdigitation lead to stronger fixation of the implant [4,5]. As discussed previously, whether a tourniquet increases bone cement penetration is controversial, with contradicting evidence [14,15]. This study represents the largest of its kind in the published literature. We demonstrated a statistically significant improvement in median total cement penetration on postoperative AP and lateral radiographs in patients undergoing TKA with tourniquet application compared to those without. Specifically, the tourniquet group exhibited a combined cement penetration of 39.14 mm on average, whereas the non-tourniquet group had 33.3 mm (p<0.01), as measured on both anteroposterior and lateral radiographs. This corroborates the findings of Sun et al. in their systematic review and meta-analysis involving 1231 patients, showing that tourniquet use enhances cement penetration during TKA [14].

Conversely, conflicting evidence from another meta-analysis by Yao et al. involving 675 patients found no significant benefits of tourniquet use in terms of cement penetration, intraoperative blood loss, or postoperative pain management [15]. This discrepancy underscores the multifaceted nature of tourniquet effects, suggesting that additional variables such as surgical technique, patient demographics, and perioperative management protocols may influence outcomes in TKA. However, the studies included in this meta-analysis were significantly smaller in size than our own.

In our study, tourniquet use was associated with a statistically significant reduction in intraoperative blood loss, evidenced by a lower drop in hemoglobin levels (-13.4 vs -15.5, p=0.03). This contrasts with earlier studies showing no significant difference [12]. However, despite this reduction in blood loss, there was no corresponding decrease in the need for blood transfusions among our patients postoperatively. This finding underscores the complex interplay of factors influencing perioperative blood management in TKA, where effective hemostasis strategies and patient-specific variables may mitigate the clinical impact of reduced blood loss associated with tourniquet use [14].

Several studies have suggested an increase in thigh pain and analgesia requirements following the use of a tourniquet [22]. However, our analysis revealed no significant difference in postoperative analgesia requirements between the tourniquet and non-tourniquet groups (68% vs 60%, p=0.19). This suggests that concerns regarding increased postoperative pain associated with tourniquet use may not be substantiated, as both groups exhibited similar needs for pain management following surgery.

The use of a tourniquet has been shown to increase hospital stay in some studies [12]. Our study demonstrated that the median length of hospital stay was marginally longer in the tourniquet group compared to the non-tourniquet group (2.8 days vs 2.5 days, p=0.48). However, this difference was not statistically significant, indicating that tourniquet use did not prolong recovery or necessitate extended hospitalization periods.

Nevertheless, this study has certain limitations, being retrospective by design and methodological constraints. There is potential for selection bias through lack of randomization. Furthermore, data collection can be a challenge due to incomplete or missing data, which can introduce information bias. Our trust operates an electronic system with readily available documentation, reducing this bias. Furthermore, causality can be difficult to establish as the exposure and outcome have already occurred and can't be manipulated. However, the inclusion of an adequate sample size and thorough statistical analysis provides a reliable set of results.

## Conclusions

Our study supports the beneficial role of tourniquet use in enhancing early postoperative cement penetration and reducing hemoglobin drop following TKA. Despite the potential positive correlation in the literature between cement penetration and long-term implant survivability, the clinical long-term implications of this improvement have not been fully determined. Hence, the decision to employ a tourniquet should be weighed against potential risks and patient-specific factors, and, as discussed earlier, the tourniquet can be deflated before closure to reduce total time and mitigate complications.

Future research endeavors should focus on conducting robust, long-term studies to elucidate the enduring effects of tourniquet use on implant survival, patient-reported outcomes, and overall healthcare utilization in TKA. By addressing these knowledge gaps, orthopedic surgeons can refine their surgical approaches and optimize patient care pathways to achieve superior outcomes in knee arthroplasty.
